# Idiopathic cutaneous immunoglobulin G/immunoglobulin M vasculitis is likely a distinct entity: A European multicentric retrospective study

**DOI:** 10.1016/j.jdin.2026.01.013

**Published:** 2026-02-04

**Authors:** Marzia Caproni, Erkan Alpsoy, Angelo Valerio Marzano, Elena Biancamaria Mariotti, Elena Del Bianco, Beatice Bianchi, Alberto Corrà, Lavinia Quintarelli, Alice Verdelli, Carlo Alberto Maronese, Cumhur I. Başsorgun, Cord Sunderkötter

**Affiliations:** a Department of Health Sciences, Rare Diseases Unit, Azienda USL Toscana Centro, Section of Dermatology, University of Florence, European Reference Network Skin Member, Florence, Italy; b Department of Dermatology, Akdeniz University, Antalya, Turkey; c Department of Dermatology, Dermatology Unit, Fondazione IRCCS Ca' Granda Ospedale Maggiore Policlinico, Milan, Italy; d Department of Pathophysiology and Transplantation, Università degli Studi di Milano, Milan, Italy; e Department of Health Sciences, Section of Dermatology, University of Florence, Florence, Italy; f Department of Pathology, Akdeniz University, Antalya, Turkey; g Department of Dermatology and Venereology, Martin Luther University Halle-Wittenberg, Halle (Saale), Germany; h Department of Dermatology and Venereology, Medical School Berlin, Berlin, Germany

**Keywords:** anaphylactoid purpura, cutaneous vasculitis, IgA vasculitis or Henoch-Schoenlein purpura, IgM-IgG vasculitis, leukocytoclastic vasculitis, non-IgA vasculitis, purpura

*To the Editor:* One group of immune complex vasculitides (IC-V) was considered to be caused by (peri)vascular deposits of large precipitating IC of immunoglobulin (Ig) M or IgG, based on the concept of serum-sickness or the Arthus-reaction, with drugs or microbes presenting the eliciting antigens.^1^^,^^2^ This IC-V was termed hypersensitivity or cutaneous leukocytoclastic vasculitis; and, lately, IgG/IgM-vasculitis (IgG/IgMV),^3^^,^^4^ to distinguish it from (1) IgA-vasculitis, given their overlapping clinical, but presumably different pathophysiologic features (hypogalactosylated IgA1 in IgAV) and (2) other IC-V associated with IgM/IgG-deposits which, however, involve special, defined IC (cryoglobulins in cryoglobulinemic-vasculitis; IgM-rheumatoid factor in rheumatoid-vasculitis, anti-C1q in hypocomplementemic urticarial vasculitis) or additional disease-related mechanisms as in systemic lupus erythematosus (SLE)-associated-vasculitis or Sjögren's syndrome-vasculitis. (Peri)vascular Ig deposits may also occur in cases of ANCA-associated vasculitides (AAV).

When hypogalactosidated IgA1 was discovered to present a major pathophysiological component in IgAV, the concept of IgG/IgMV had not been extensively pursued. Therefore, this multicenter retrospective study by the EADV-Vasculitis-Taskforce investigated the existence and frequency of idiopathic IgM/IgGV.

Data were collected from 2017 until May 2024 in the Dermatology Departments of Florence, Milan, and Antalya, where vasculitis cases were documented according to a standard operating procedure previously consented by the EADV-Taskforce. Inclusion criteria were: (1) palpable purpura; (2) histologically confirmed leukocytoclastic vasculitis with (peri)vascular IgM and/or IgG, but without IgA (immunofluorescence analyzed by the skin-immunopathology-laboratory, Florence); and (3) follow-up ≥18 months.

Among all archived small-vessel-vasculitides 90 cases were retrieved with (peri)vascular deposition of IgG or IgM, but no IgA (Table I). Of these, 45 had to be excluded because data necessary to indicate or exclude other forms of vasculitis were missing (Table II), and 2, because a second biopsy had revealed presence of (peri)vascular IgA. Of the remaining 43 cases, 7 revealed another defined vasculitis: 3 AAV, 3 vasculitis in Sjögren's syndrome and 1 in SLE. Consequently, 36 of the 43 patients fulfilled the complete criteria for idiopathic IgG/IgMV. None of these cases exhibited systemic involvement and of 33 patients followed up until August 2025, only 1 had relapsed.

**Table I:** Results of direct immunofluorescence

DIF results	Florence	Milan	Antalya	
Total	20	11	5	
(peri)vascular IgG	0	0	3	3 (8.33% of all idiopathic IgG/IgMV)
(peri)vascular IgM	16	11	2	29 (80.6%)
both (peri)vascular IgG and IgM	4	0	0	4 (11.1%)

*IgG*, Immunoglobulin G; *IgM*, immunoglobulin M.

**Table II:** Idiopathic IgM/IgG*V* cases during the study period related to all histologically confirmed small vessel vasculitides, and number of vasculitides with exclusively (peri)vascular IgG/IgM-deposits prior to exclusion of those with incomplete data and after exclusion of those whose data indicated presence of another form of IC-vasculitis or of AAV. Data shown for each of the 3 centers

	Florence	Milan	Antalya	Total
Number of histologically confirmed small vessel vasculitides	155	126	55	336
Confirmed IC-V with exclusively IgG/IgM deposits in initial biopsy (with complete or incomplete sets of laboratory data)	50 (32.3%)	27 (21.4%)	13 (23.6%)	90 (26.7% of all histologically confirmed small vessel vasculitides)
Confirmed IC-V with exclusively IgG/IgM deposits in initial biopsy and with complete datasets	24 (15.4%)	14 (11.1%)	7 (12.7%)	45 (13.4%)
Idiopathic IgM/IgGV (after exclusion of cases with other defined IC-V such cryoglobulinemic, Sjögren's and SLE vasculitis or of cases with AAV and of cases with IgA in subsequent biopsies)	20 (12.9%)	11 (8.7%)	5 (9.0%)	36 (10.7%)

*AAV*, ANCA-Associated vasculitides; *IC-V*, immune complex vasculitides; *IgA*, immunoglobulin A; *IgG*, immunoglobulin G; *IgM*, immunoglobulin M.

These findings would comply with the existence of idiopathic IgG/IgMV as a distinct entity. They also indicate that it is likely infrequent and benign. Confirmation of its diagnosis, however, requires the described comprehensive evaluation to exclude and to not overlook another form of vasculitis, such as AAV, Sjögren's syndrome-vasculitis or SLE-vasculitis—as observed in this cohort—or cryoglobulinemic or rheumatoid-vasculitis as shown in other reports.^2^, ^3^, ^4^, ^5^ Previous immunofluorescence studies on IC-V have revealed considerable variability in frequency of exclusively (peri)vascular IgM/IgG, and often lacked the complete clinical and pathological data necessary to sufficiently differentiate between different IC-V (reviewed in^5^)—a deficiency that even in our cohort forced exclusion of several initially identified cases.

Other reasons for (exclusively) IgG/IgM deposits could be (1) opsonization of necrotic endothelium for clearance by phagocytes, which, however, would presumably occur later and not only around clearly discernible vascular structures, or (2) failure to detect IgA, which may occur (as in the 2 cases we excluded, because a second biopsy had revealed IgA), but which is unlikely to have occurred in all our biopsies.

In conclusion, it is clinically relevant to differentiate between IgG/IgMV and other IC-V.

## Conflicts of interest

None disclosed.
